# Supplementary material for: Streamlined CRISPR-based assays for detection and subtyping of H5 and H7 avian influenza
Source: iScience. 2026 Sep 18;29(10):117556. doi: 10.1016/j.isci.2026.117556 (PMC13626927; doi:10.1016/j.isci.2026.117556)
Supplement: Supplementary file 1 [file mmc1.pdf]

iScience, Volume *29*

Supplemental information

Streamlined CRISPR-based assays for detection and subtyping of H5 and H7 avian influenza

Yujia Huang, Andrew Guo, Gordon Adams, Jacob E. Lemieux, and Cameron Myhrvold

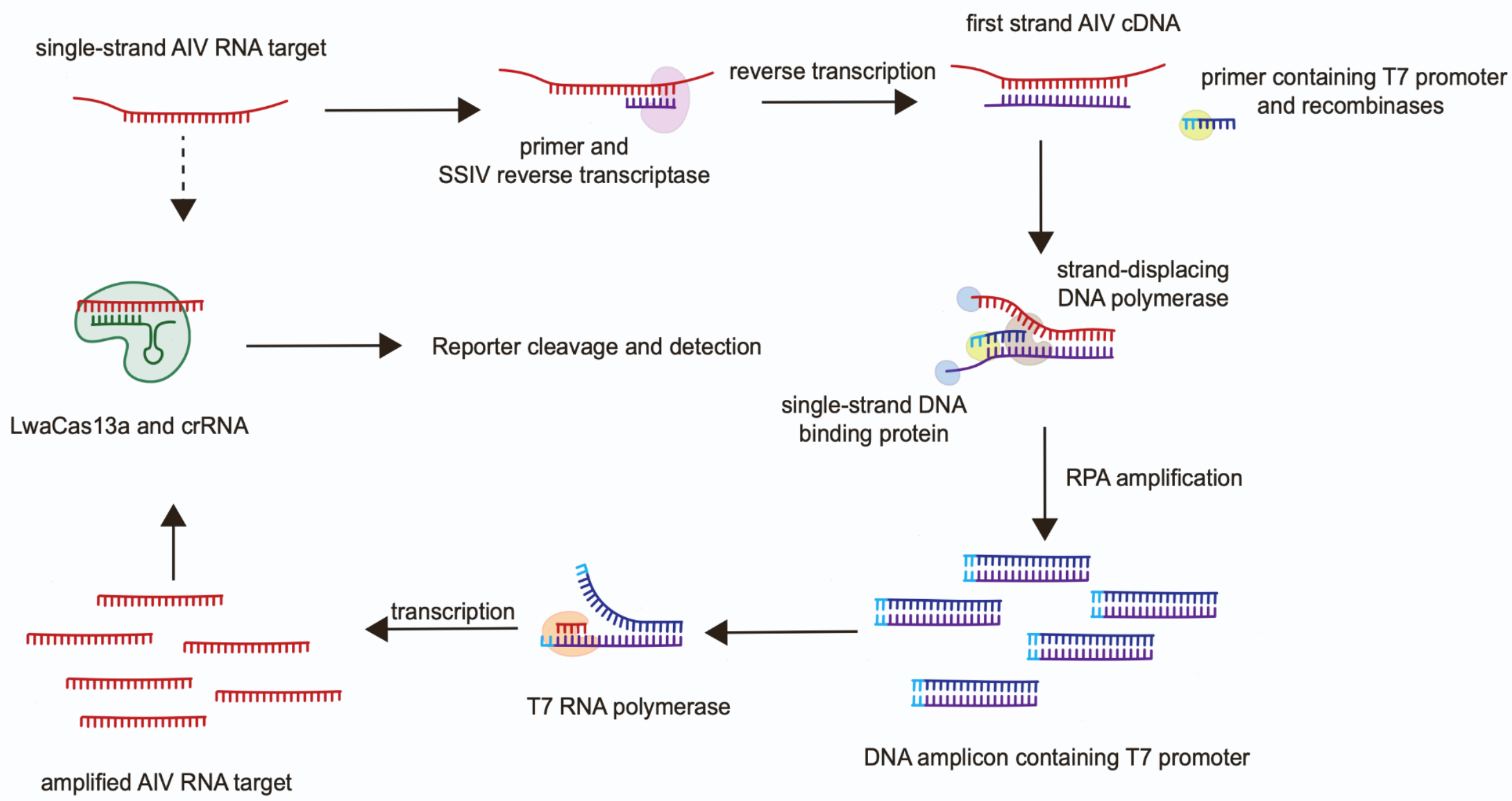

Supplementary Figure 1. Schematic of target amplification of avian flu SHINE. Single-stranded AIV RNA target is reverse transcribed by primer and SSIV into cDNA, then it gets amplified through RPA amplification. T7 RNA polymerase then further transcribes the DNA amplicon into AIV RNA targets, which can be detected by LwaCas13a. All reactions depicted happen in one pot.

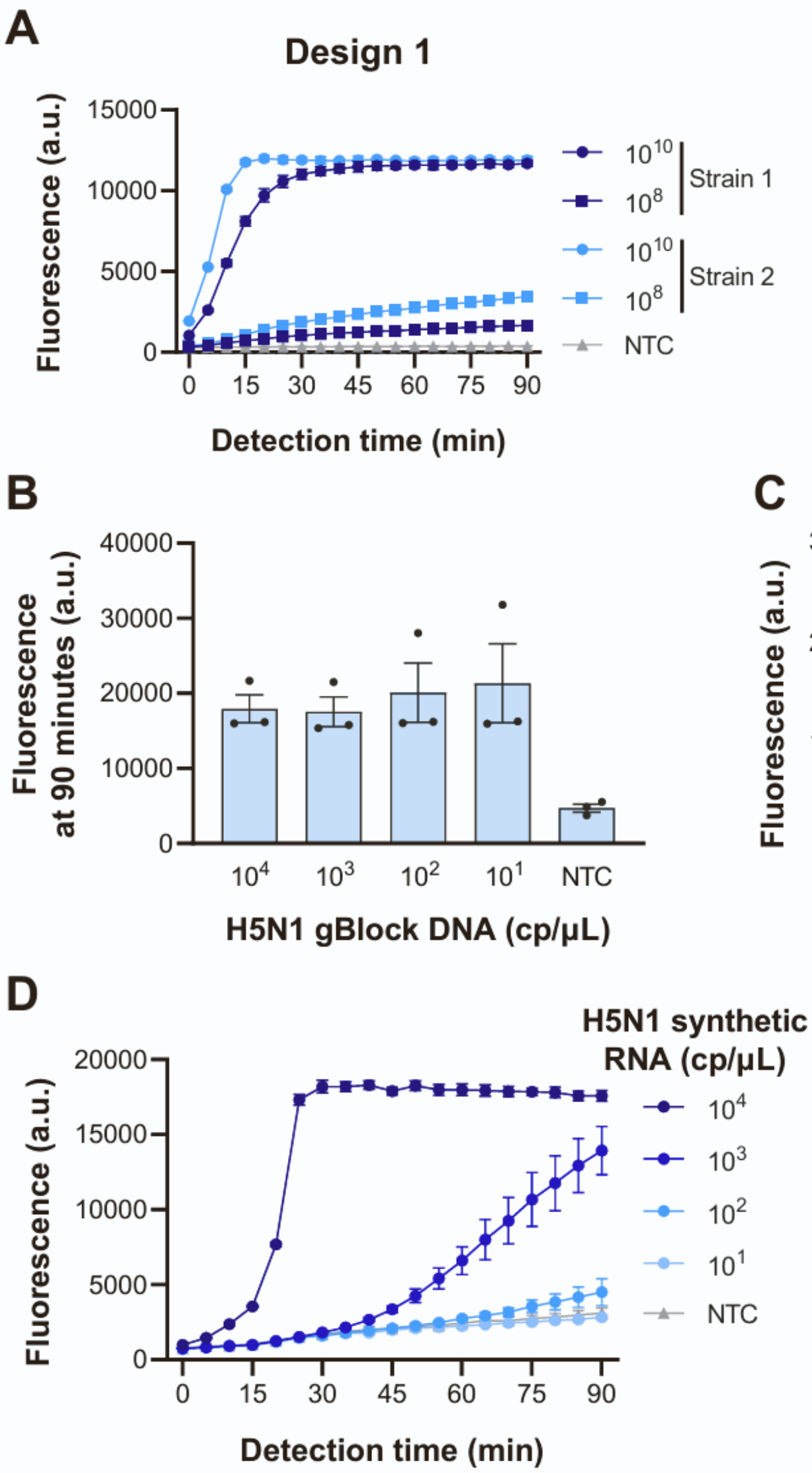

Supplementary Figure 2. Kinetics of prototype H5 SHINE assay. A) Kinetics from Figure 1C, separated by design. **B)** Fluorescence readout of H5N1 SHINE assays on a serial dilution of double-stranded DNA templates that encoded the entire H5 segment. **C)** Kinetics from Supplementary Figure 1B. **D)** Kinetics from Figure 1D. Timepoints represent mean fluorescence ± standard deviation of 3 technical replicates. NTC: no target control.

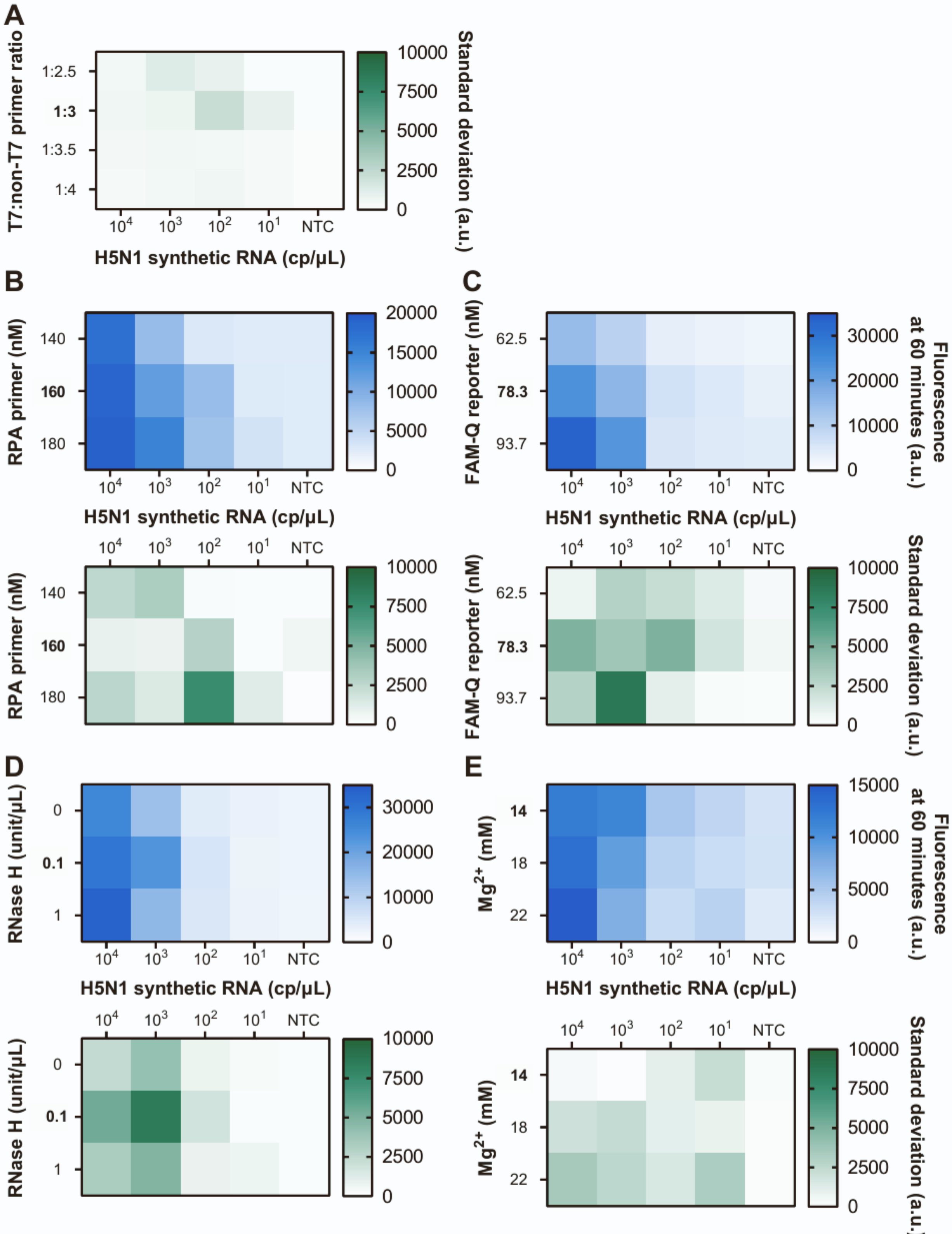

A
T7:non-T7 primer ratio
1:2.5
1:3
1:3.5
1:4
10^4
10^3
10^2
10^1
NTC
H5N1 synthetic RNA (cp/μL)
Standard deviation (a.u.)
10000
7500
5000
2500
0
B
RPA primer (nM)
140
160
180
20000
15000
10000
5000
0
C
FAM-Q reporter (nM)
62.5
78.3
93.7
Fluorescence at 60 minutes (a.u.)
30000
20000
10000
D
RNase H (unit/μL)
0
0.1
1
E
Mg2+ (mM)
14
18
22
15000

Supplementary Figure 3. Optimization of H5 SHINE assay (heatmap). Heatmap showing the H5 SHINE assay performance at conditions with different **A)** RPA primer concentration, **B)** FAM-Q reporter concentration, **C)** RNase H concentration, **D)** Mg^{2+} concentration. Blue represents mean fluorescence and green represents standard deviation of 3 technical replicates for each cell in heatmap. Bolded conditions (RPA primer at 160nM, FAM-Q reporter at 78.3nM, RNAse H at 0.1units/μL, Mg^{2+} at 18mM) were selected for further development. NTC: no target control.

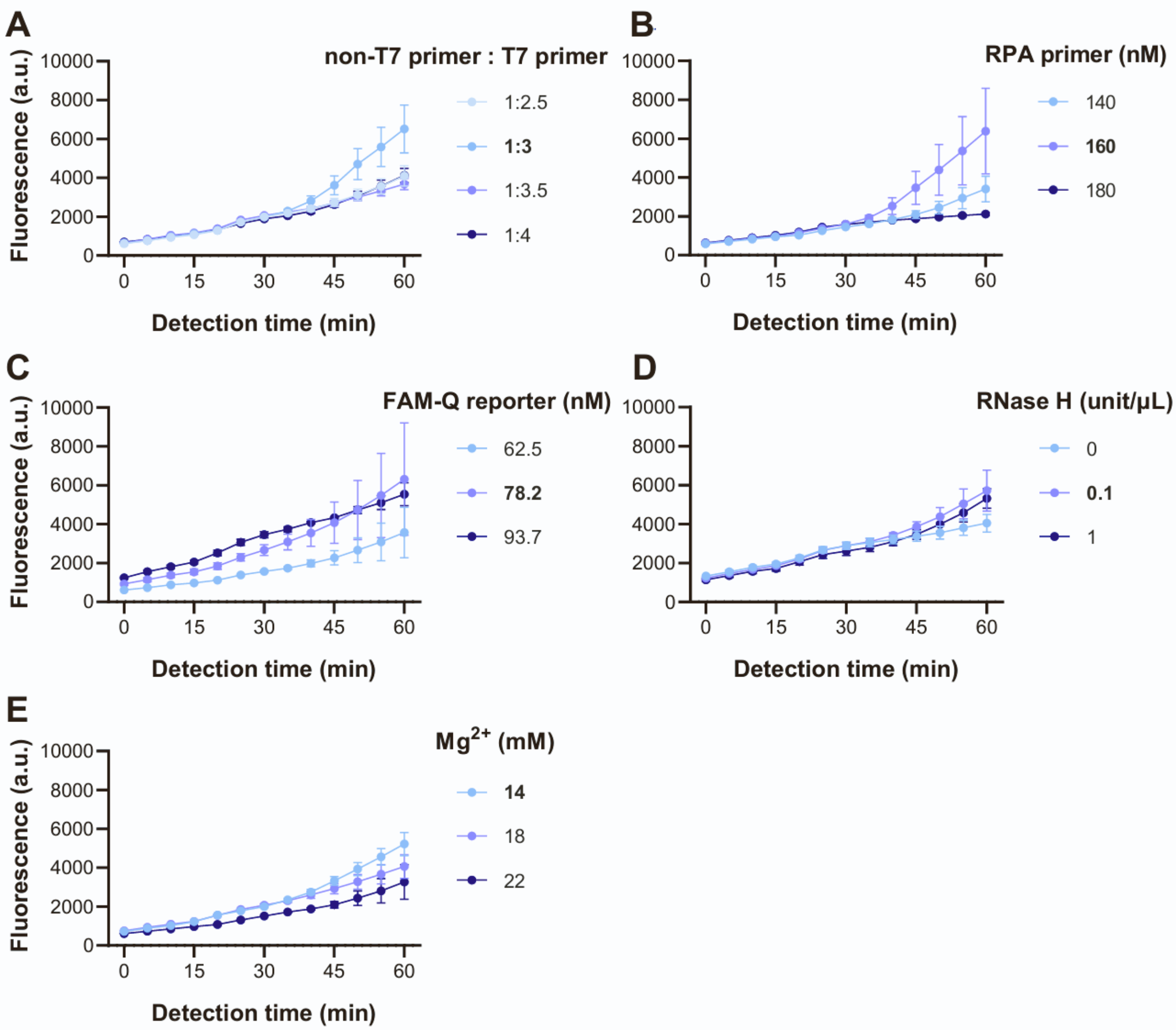

Supplementary Figure 4. Kinetics of H5 SHINE assay optimization. Kinetics from **A)** Figure 2A. **B)** Supplementary Figure 2B. **C)** Supplementary Figure 2C. **D)** Supplementary Figure 2D. **E)** Supplementary Figure 2E. All reactions contain H5N1 synthetic RNA at 10^2 copies/μL. Timepoints represent mean fluorescence ± standard deviation of 3 technical replicates. Bolded conditions (non-T7 primer : T7 primer at 1:3 ratio, RPA primer at 160nM, FAM-Q reporter at 78.3nM, RNAse H at 0.1units/μL, Mg^{2+} at 18mM) were selected for further development. Timepoints represent mean fluorescence ± standard deviation of 3 technical replicates. NTC: no target control.

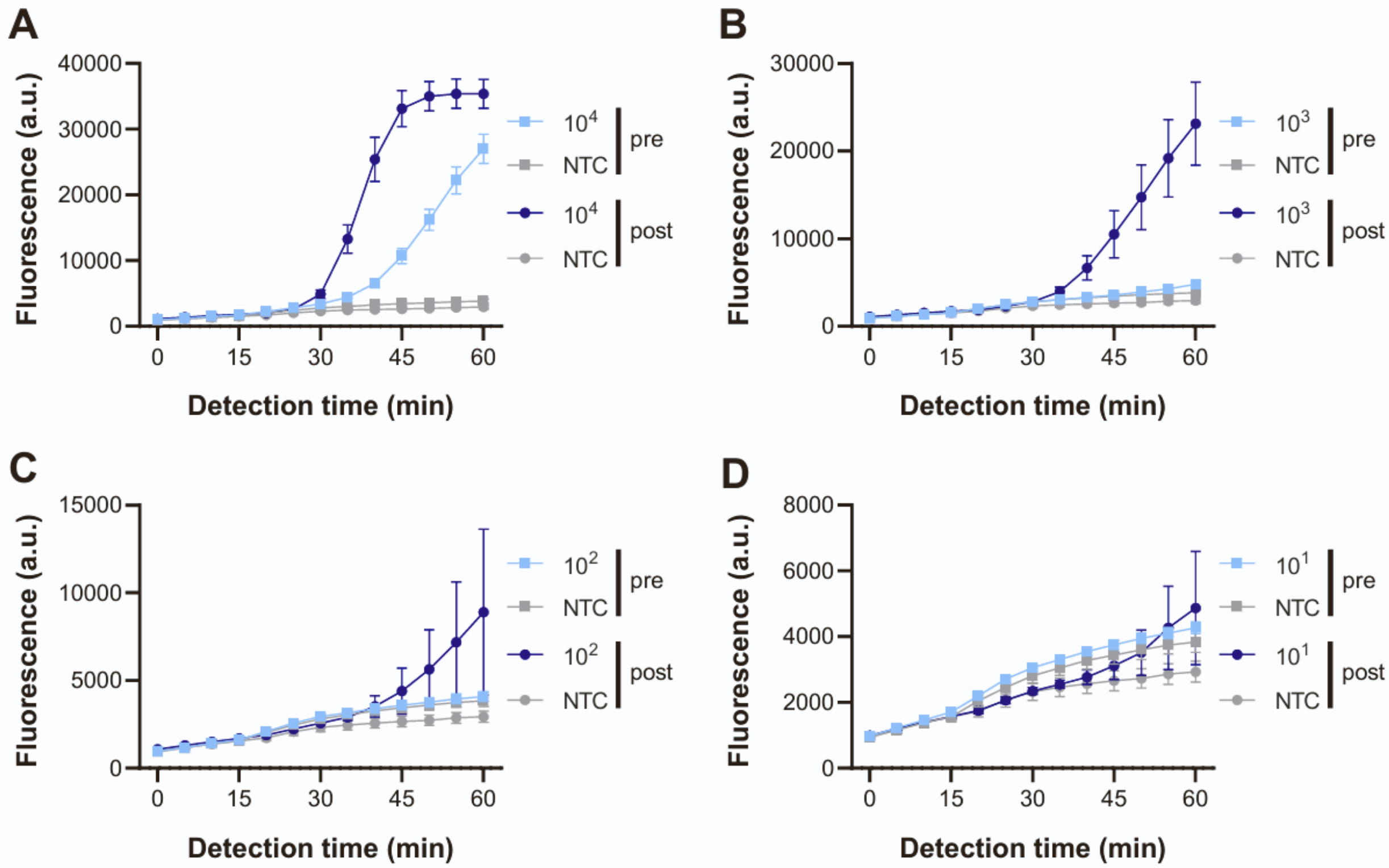

Supplementary Figure 5. Kinetics of pre- vs post-optimization. Kinetics from Figure 2B, separated by H5N1 synthetic RNA input concentration at **A)** 10^4 cp/µL, **B)** 10^3 cp/µL, **C)** 10^2 cp/µL, **D)** 10^1 cp/µL, Timepoints represent mean fluorescence ± standard deviation of 3 technical replicates. NTC: no target control, is shared in all panels.

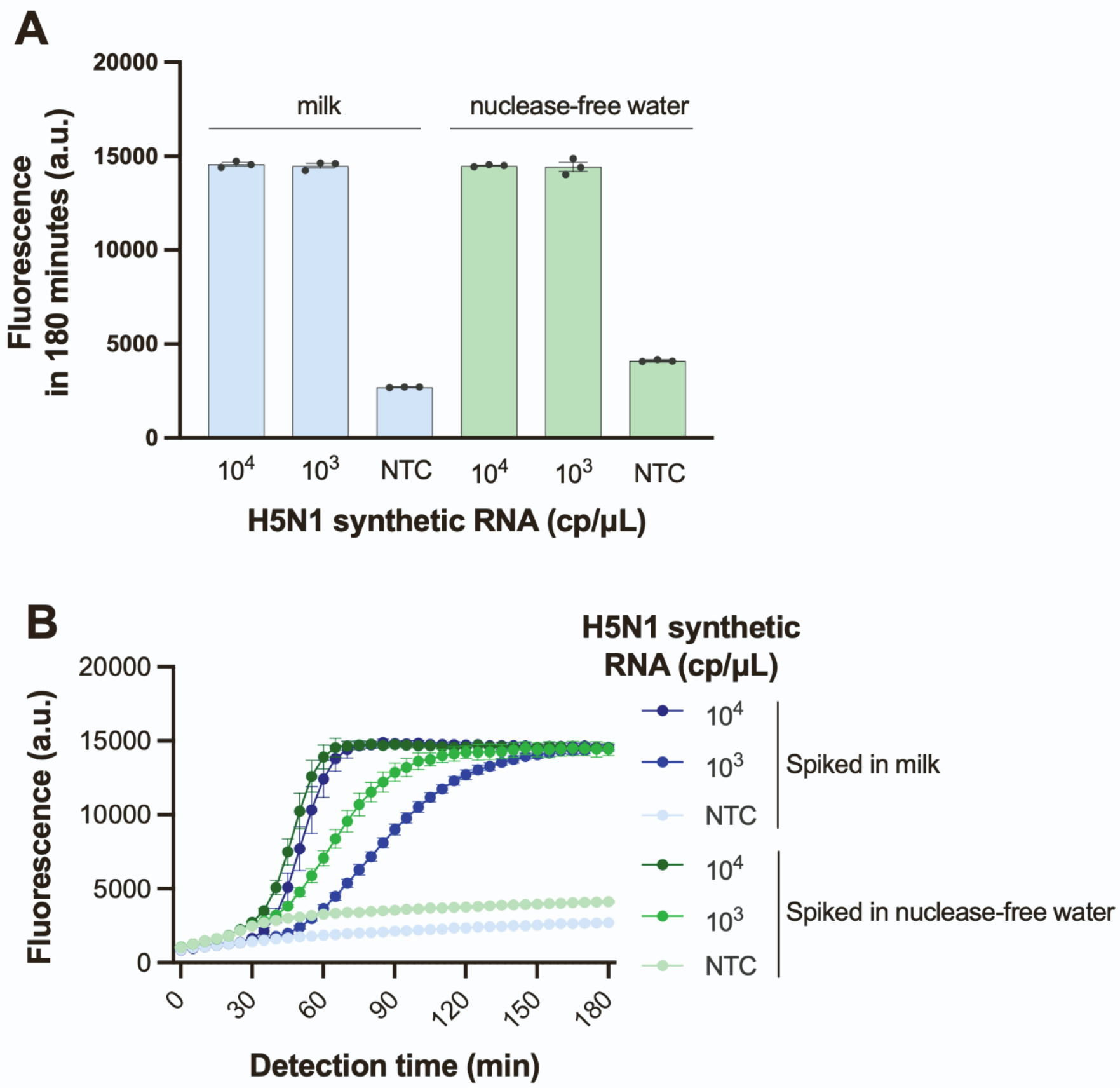

Supplementary Figure 6. Detection of H5N1 in mock samples in milk using SHINE-H5. A) Comparison of fluorescence readout of H5N1 SHINE assays on H5N1 synthetic RNA spiked in milk and nuclease-free water **B)** Kinetics from Supplementary Figure 6A, milk samples showed slower kinetics, but reached similar final readout compared to nuclease-free water.

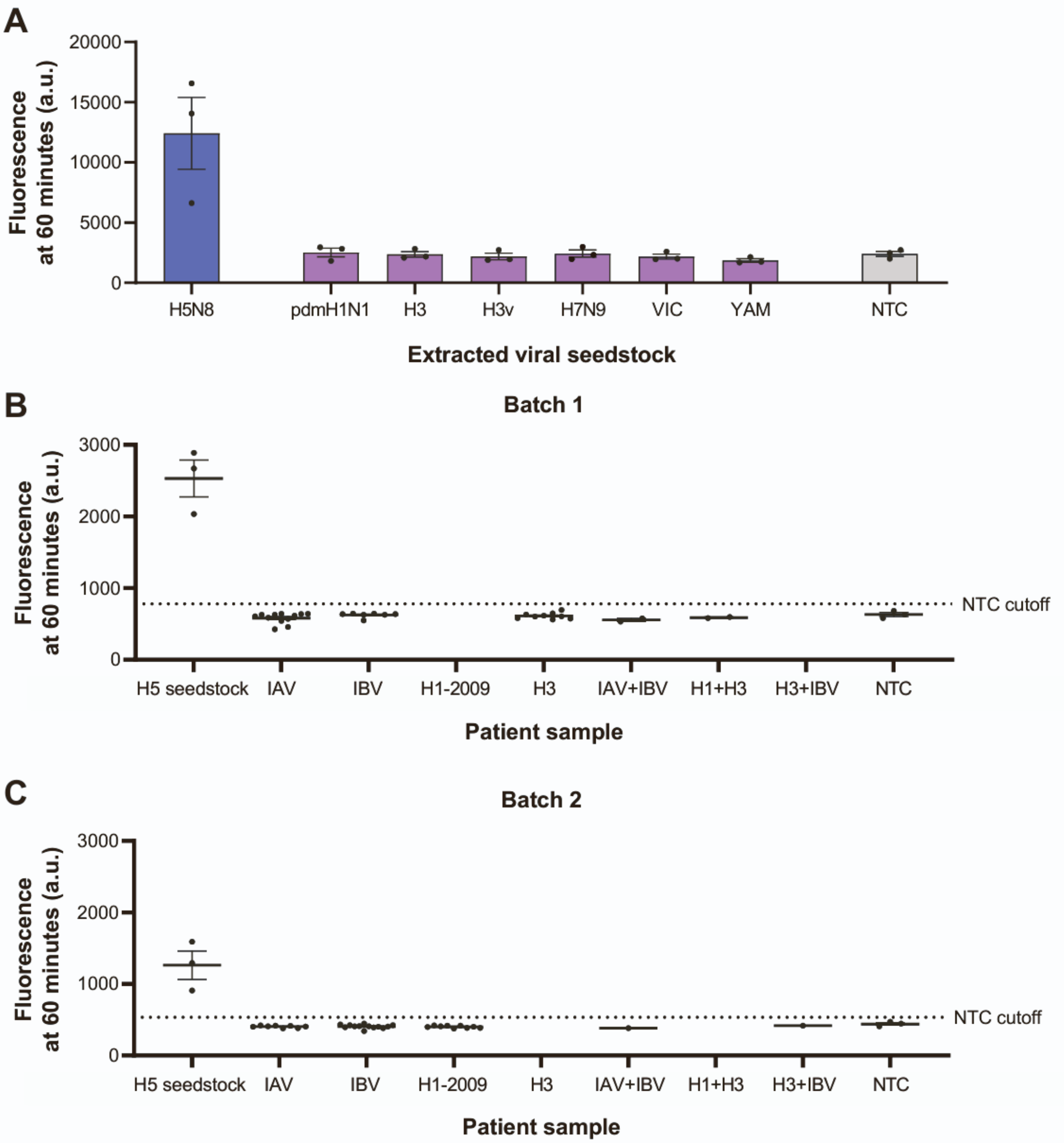

Supplementary Figure 7. SHINE-H5 specificity test. A) Cross-reactivity panel for SHINE-H5 against various seasonal influenza viral seedstocks. See Supplementary Table 2 for strain information. **B) C)** Fluorescence readout of optimized SHINE-H5 on RNA extracted from H5N1 viral seedstock (10^2 cp/µL) and from clinical NP swabs characterized by NAAT (BioFire) or qPCR. Samples were divided into two batches for testing. **B)** Batch 1. **C)** Batch 2. Each dot shows the mean of 3 technical replicates of a specific patient sample after 60 minutes. Cutoff is set as mean + three times standard deviation of 3 technical replicates of NTC (no target control). See Supplementary Table 3 for detailed patient information and qPCR/NAAT/SHINE readout.

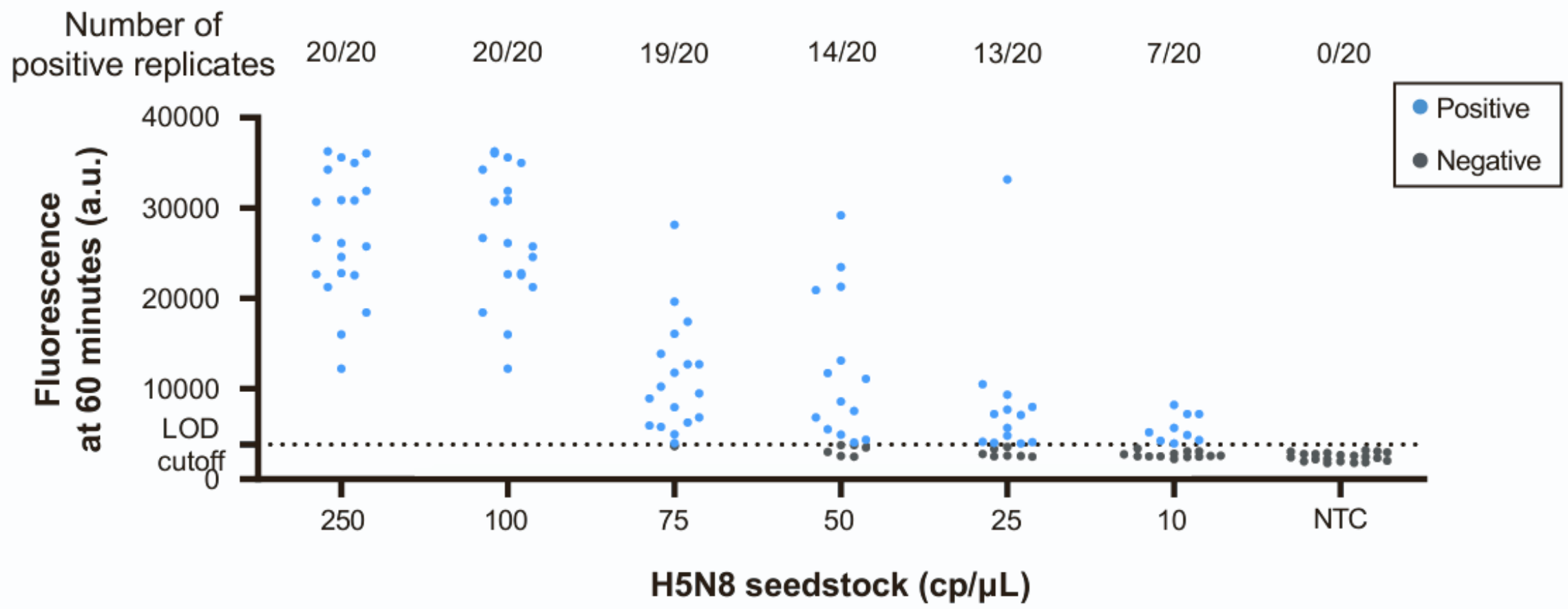

Supplementary Figure 8. SHINE-H5 LOD. Individual fluorescence values from Figure 2D. Cutoff is set as mean + three times standard deviation of 20 technical replicates of NTC (no target control).

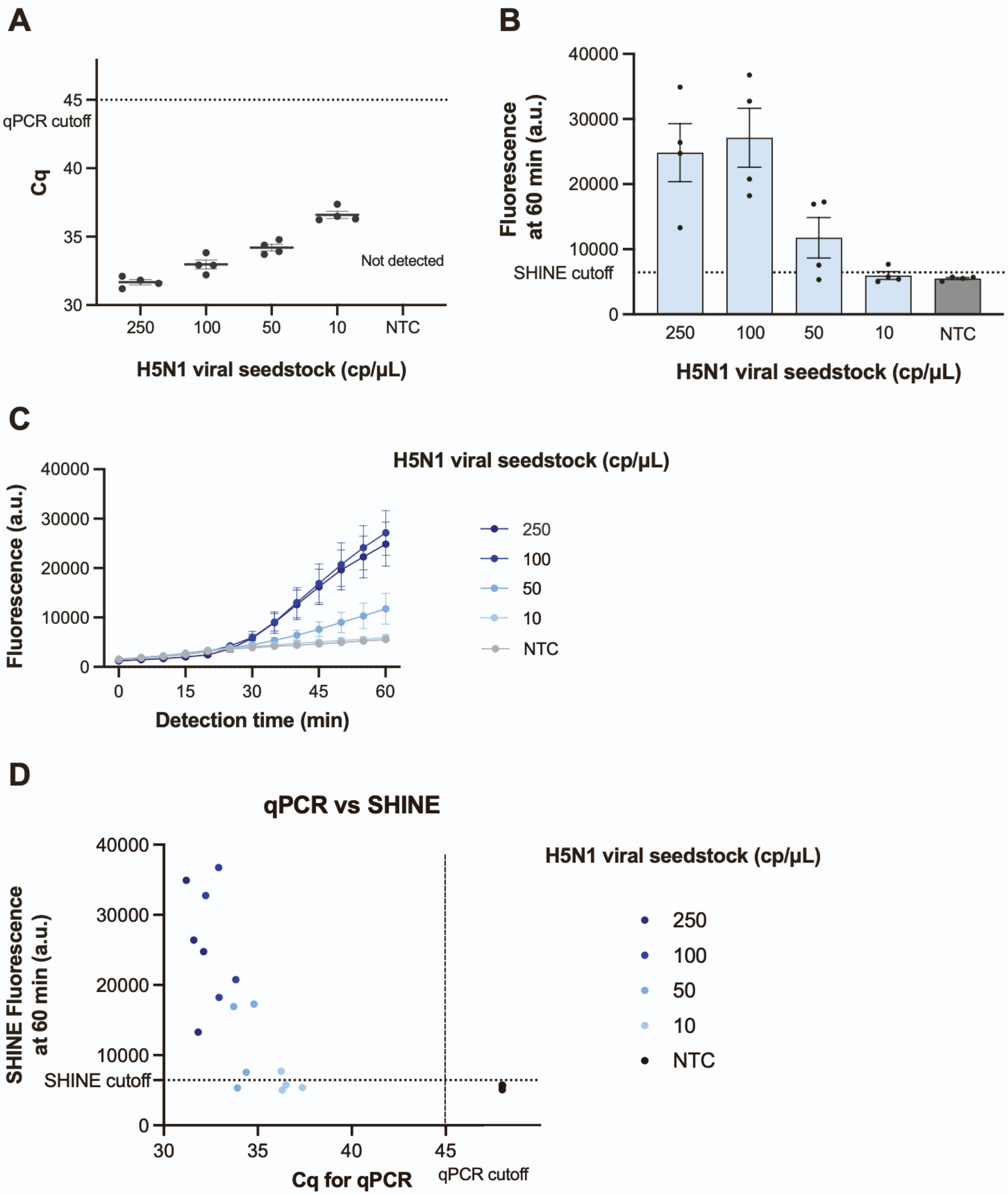

Supplementary Figure 9. Comparison of SHINE and qPCR.; **A)** Cq value of qPCR on RNA extracted from H5N1 viral seedstock; **B)** Fluorescence readout of H5N1 SHINE assays on RNA extracted from H5N1 viral seedstock. **C)** Kinetics from Supplementary Figure 9B. **D)** Concordance between RT-qPCR and SHINE-H5 on H5 viral seedstock; NTC: no target control. In **A**, **B** and **C**, values are mean fluorescence ± standard deviation of 4 technical replicates. The SHINE cutoff is the mean fluorescence of NTC + 3×standard deviation and the qPCR cutoff is a Cq value of 45.

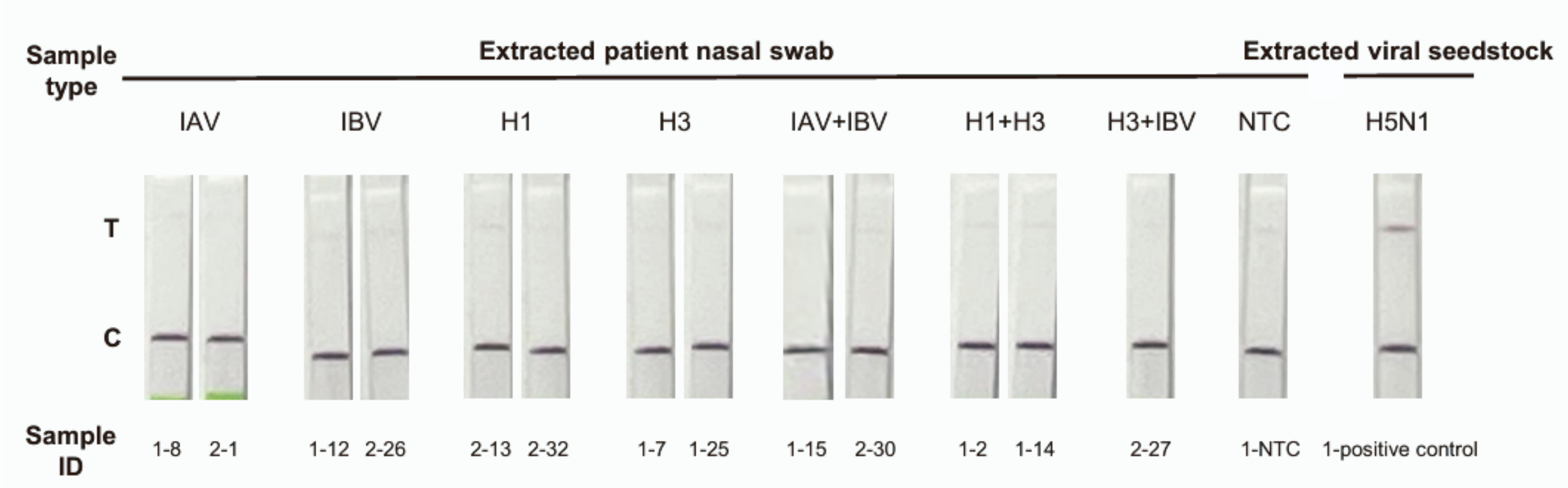

Supplementary Figure 10. SHINE-H5-LFA specificity. Paper-based colorimetric readout of SHINE-H5-LFA on RNA extracted from H5N1 viral seedstock (10^2 cp/µL) and from clinical NP swabs characterized by NAAT (BioFire) or qPCR after 60-minute incubation. NTC: no target control. See Supplementary Table 3 for detailed patient information and qPCR/NAAT/SHINE readout.

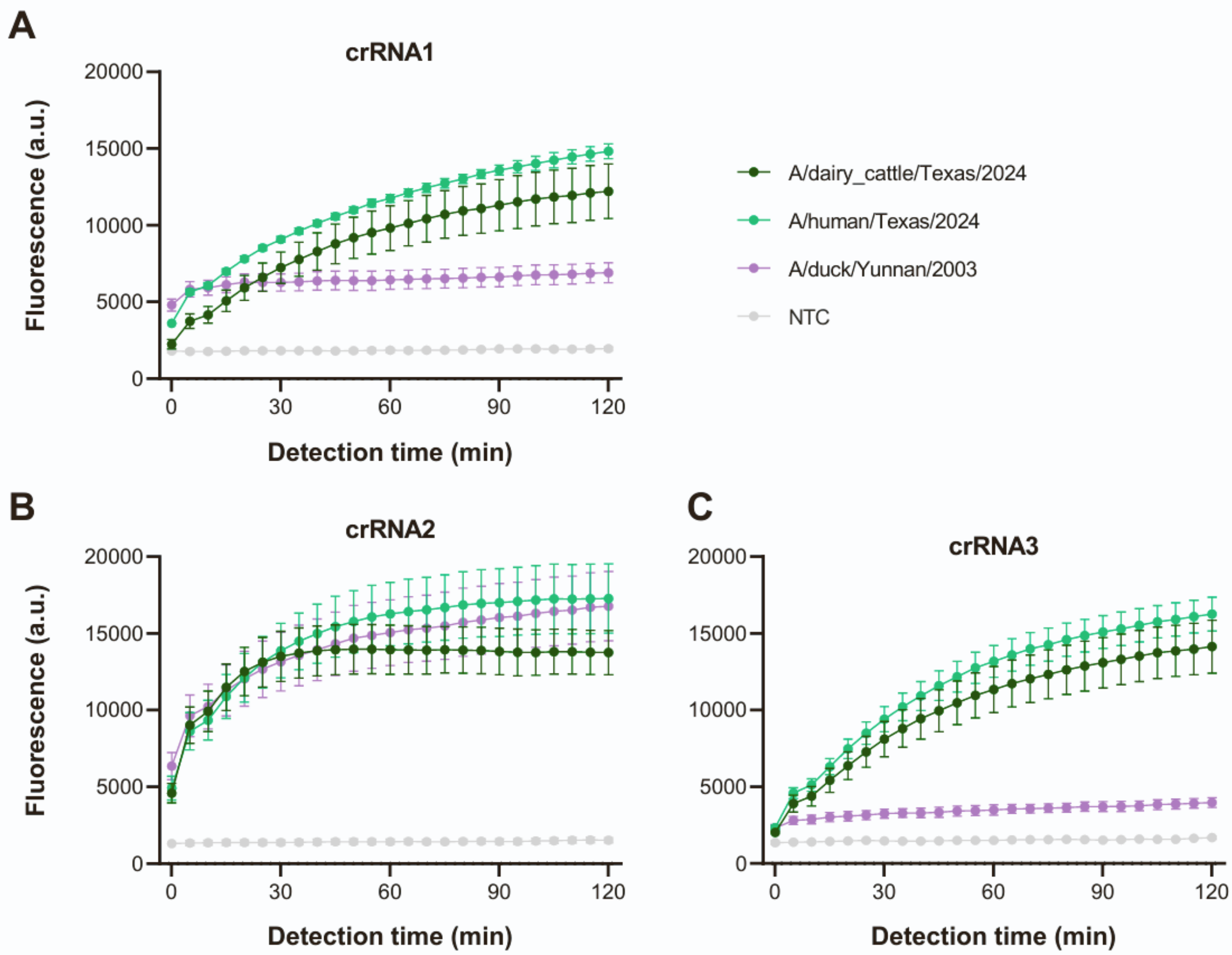

Supplementary Figure 11. Kinetics of clade 2.3.4.4b A(H5N1) SHINE crRNA screening. Kinetics from Figure 3C, separated by crRNA input **A)** crRNA 1, **B)** crRNA 2, **C)** crRNA 3. NTC: no target control.

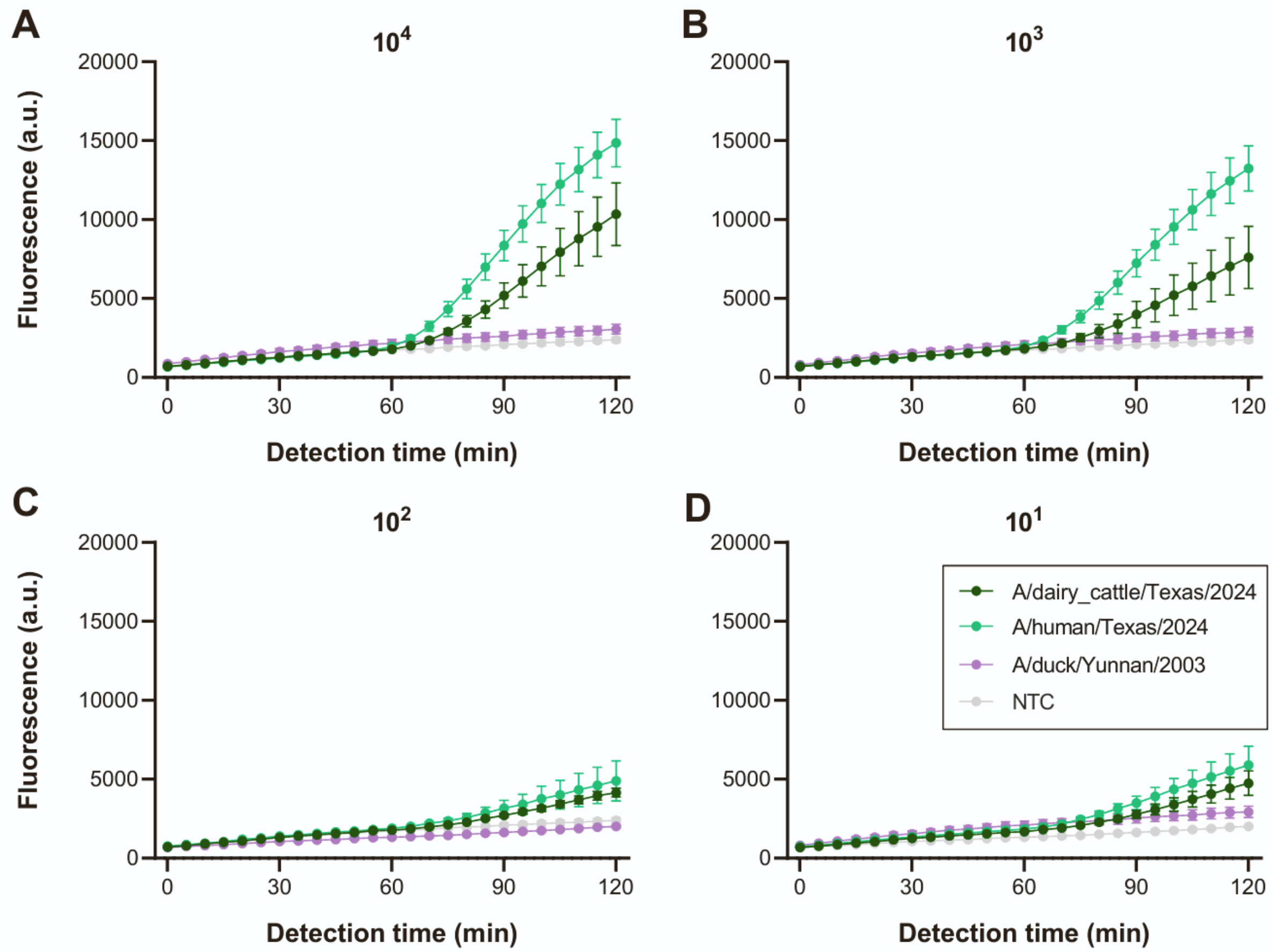

Supplementary Figure 12. Kinetics of SHINE-H5-CS. Kinetics from Figure 3D, separated by synthetic H5 RNA input concentration at **A)** 10^4, **B)** 10^3, **C)** 10^2, **D)** 10^1. Timepoints represent mean fluorescence ± standard deviation of 3 technical replicates. NTC: no target control.

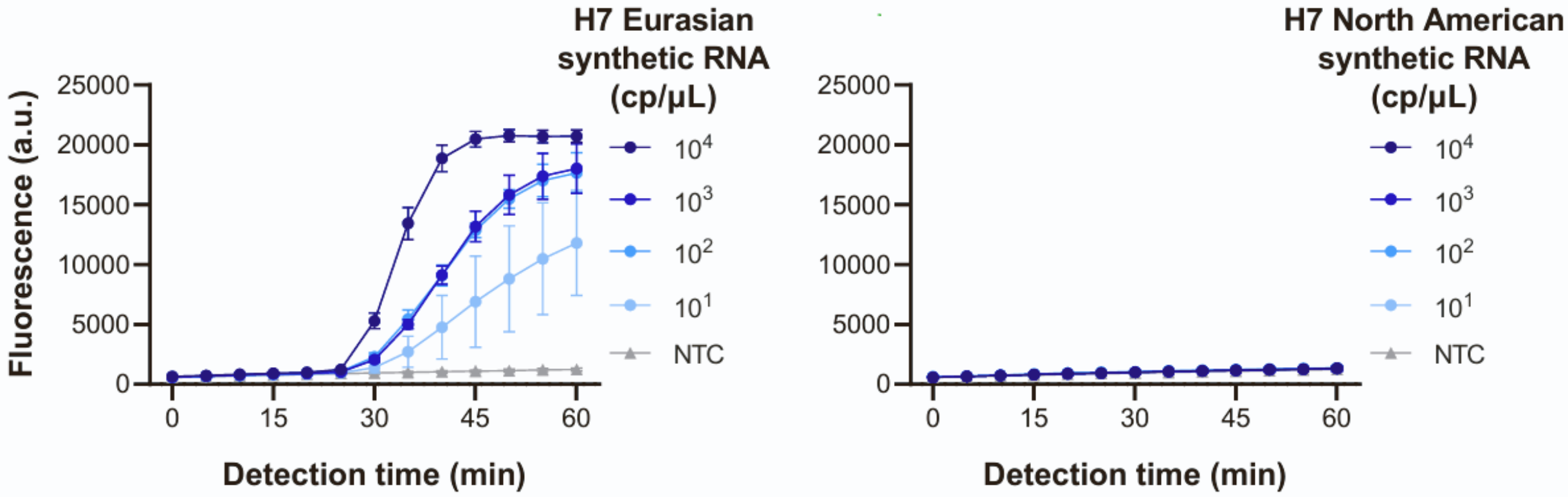

Supplementary Figure 13. Kinetics of SHINE-H7-Eurasian. Kinetics from Figure. 4C, separated by H7 RNA input type. Timepoints represent mean fluorescence ± standard deviation of 3 technical replicates. NTC: no target control.

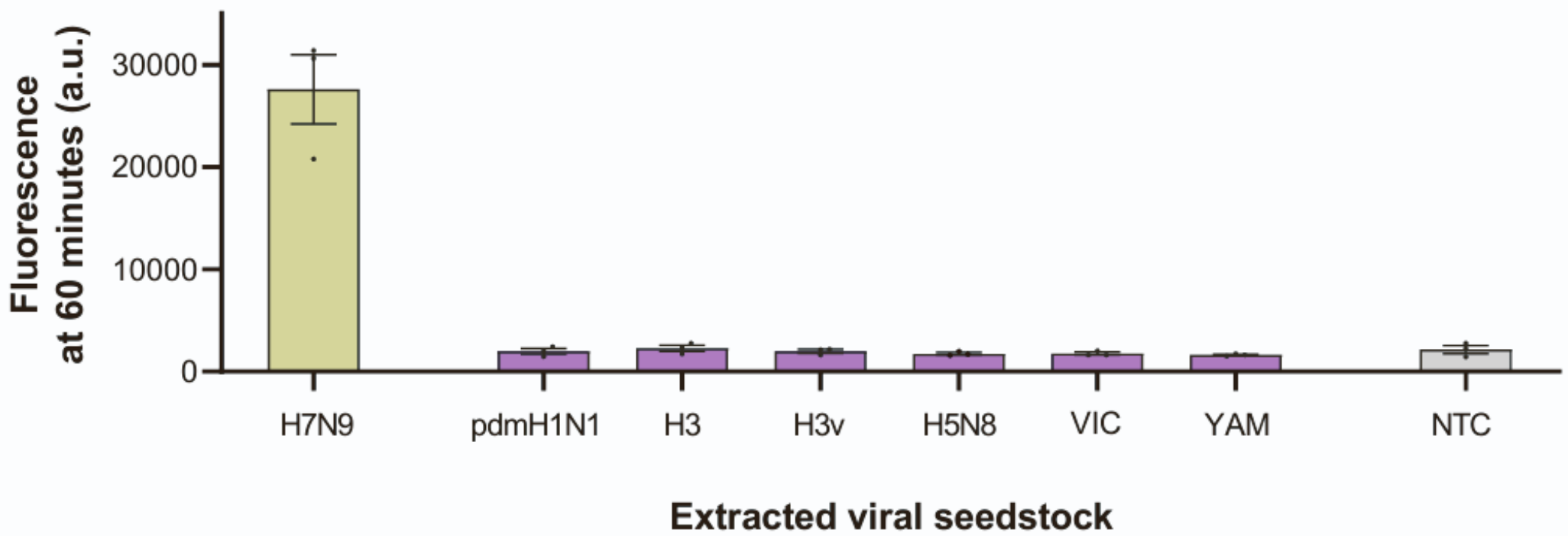

Supplementary Figure 14. SHINE-H7-Eurasian specificity test. Cross-reactivity panel for SHINE-H7-Eurasian against various seasonal influenza viral seedstocks. See Supplementary Table 3 for strain information.

Supplementary Table 1. Summary of current CRISPR-Dx for AIV detection.

Reference	Combined (Y/N)	Target gene	Input	Subtyping/ clade typing	Cas protein(s)	Sample-to-answer time (min)	Analytical sensitivity (cp/µL)		Off-target seedstocks tested	Clinical Sensitivity (%)	Clinical Specificity (%)
							Fluorescence	LFA			
28	N	HA	RNA	H5	LwaCas13a	50	N.D.	1	H3, H7, H9, H10-AIV, IBV, NDV, RVA and DAstV	81.6	100
29	N	M	cDNA	No	LwaCas13a	N.D.	69	690	NDV, IBV, IBDV	N.D.	N.D.
30	N	NP	cDNA/ plasmid	No	LwaCas13a	60	N.D.	1	NDV, IBV, ILTV	N.D. §	N.D.
31	N	M, NP	RNA	No	LbaCas12a	150	6.7 (M) ,12 (NP)	67 - (M) , 1.2×10^4 (NP)	NDV, IBV, IBDV‡	98.1	96
32	N	HA	RNA	H9N2	LwaCas13a	60-90†	1	N.D.	H5N1 AIV, H1N1 AIV, NDV, IBDV, and IBV	N.D.	N.D.
33	N	HA	RNA	H5	LbaCas12a	60-180	1.9	1.9×10^3	16 subtypes of AIVs (H1-H16), NDV, IBV, IBDV	93.75	84.84
34	N	HA	RNA	H5	LbaCas12a	60	100 EID_{50}/mL	1000 EID_{50}/mL	H9N2, H1N1, H3N2, IBV, H1N5, H7N5, H6N2, H11N2, H10N7	80	100
35	N	M, HA	RNA/plasmid*	AIV, H5, H7, H9	LwaCas13a	50	1	10	H1, H3, H6, H10, NDV, IBV, IBDV, DTMUV	93.1 (Fluorescence), 92.8 (LFA)	94.1 (Fluorescence), 88.2 (LFA)
This study	Y	HA	RNA	H5, 2.3.4.4b A (H5N1), H7 Eurasian	LwaCas13a	60	121.7	25	H3, H7N9, pdmH1N1, VIC, YAM	N.D.	100¶

* Sensitivity is defined with plasmid input, other assays used RNA input

† estimated through kinetics

‡ compared to in vitro transcribed M/NP gene of these viruses

§ 98.75% coincidence rate compared to agarose gel after PCR

¶ compared to human samples infected with seasonal IAV

Acronyms: Newcastle disease virus (NDV), Infectious bronchitis virus (IBV), Infectious bursal disease virus (IBDV), Duck Tambous Virus (DTMUV)

N.D.: not determined

Supplementary Table 2. Strain information for cross-reactivity panel for Figure 3E, Supplementary Figure 5A, 11.

Sequence name	HA segment origin
pdmH1N1	A/Michigan/45/2015
A/H3	A/Singapore/INFIMH-16-0019/2016
A/H3 Variant	A/Ohio/28/2016
A/H5N1	A/gyrfalcon/Washington/41088-6/2014
A/H7N9	A/HongKong/125/2017-IDCDC-RG33A
B/VIC	B/Colorado/06/2017
B/YAM	B/Phuket/3073/2013

Supplementary Table 3. Patient sample information for Figure 2C, Supplementary Figure 5B, 5C, 7.

Sample ID	Target 1	Target 2	Target 3	CT FLU B	CT FLU A1	CT FLU A2	Average SHINE fluorescence (a.u.)	Normalized fluorescence
1 - positive control*	N/A	N/A	N/A	N/A	N/A	N/A	2529.7	1.000
1 - 1	INFLUENZA A	INFLUENZA B	0	24.5	20.2	23.7	579.0	0.229
1 - 2*	INFLUENZA A	INFLUENZA A H1-2009	INFLUENZA A H3	0	0	0	596.7	0.236
1 - 3	INFLUENZA A	INFLUENZA A H3	0	0	0	0	574.3	0.227
1 - 4	INFLUENZA A	INFLUENZA A H3	0	0	0	0	602.0	0.238
1 - 5	INFLUENZA B	0	0	20.1	0	0	628.3	0.248
1 - 6	INFLUENZA A	INFLUENZA A H3	0	0	0	0	615.0	0.243
1 - 7*	INFLUENZA A	INFLUENZA A H3	0	0	0	0	562.3	0.222
1 - 8*	INFLUENZA A	0	0	0	25.8	27.3	587.7	0.232
1 - 9	INFLUENZA A	0	0	0	31.2	34.6	604.7	0.239
1 - 10	INFLUENZA A	0	0	0	22	23.4	458.7	0.181
1 - 11	INFLUENZA A	INFLUENZA A H3	0	0	0	0	632.0	0.250
1 - 12*	INFLUENZA B	0	0	28	0	0	548.7	0.217
1 - 13	INFLUENZA A	0	0	0	21.1	21.8	629.7	0.249
1 - 14*	INFLUENZA A	INFLUENZA A H1-2009	INFLUENZA A H3	0	0	0	580.0	0.229
1 - 15*	INFLUENZA A	INFLUENZA B	0	11.8	34.6	0	532.7	0.211
1 - 16	INFLUENZA B	0	0	21.1	0	0	643.0	0.254
1 - 17	INFLUENZA A	0	0	0	31.1	31.9	614.3	0.243
1 - 18	INFLUENZA A	0	0	0	25.4	26.6	542.3	0.214
1 - 19	INFLUENZA A	0	0	0	27.4	28.7	637.3	0.252
1 - 20	INFLUENZA B	0	0	38.8	0	0	636.3	0.252
1 - 21	INFLUENZA B	0	0	32.2	0	0	637.3	0.252
1 - 22	INFLUENZA A	0	0	0	22.4	24.1	627.3	0.248

1 - 23	INFLUENZA A	INFLUENZA A H3	0	0	0	0	695.7	0.275
1 - 24	INFLUENZA A	INFLUENZA A H3	0	0	0	0	604.7	0.239
1 - 25*	INFLUENZA A	INFLUENZA A H3	0	0	0	0	581.3	0.230
1 - 26	INFLUENZA A	0	0	0	20.8	22.1	424.7	0.168
1 - 27	INFLUENZA A	0	0	0	21.6	23.3	569.7	0.225
1 - 28	INFLUENZA A	INFLUENZA A H3	0	0	0	0	647.7	0.256
1 - 29	INFLUENZA B	0	0	35.1	0	0	643.3	0.254
1 - 30	INFLUENZA B	0	0	33.7	0	0	629.3	0.249
1 - 31	INFLUENZA A	0	0	0	25.7	27.1	640.7	0.253
1 - 32	INFLUENZA A	0	0	0	34.8	34.7	642.3	0.254
1 - NTC*	N/A	N/A	N/A	N/A	N/A	N/A	631.0	0.249
2 - positive control	N/A	N/A	N/A	N/A	N/A	N/A	1262.3	1.000
2 - 1*	INFLUENZA A	0	0	0	33.5	33.2	383.3	0.304
2 - 2	INFLUENZA B	0	0	33.2	0	0	392.7	0.311
2 - 3	INFLUENZA B	0	0	30.5	0	0	382.0	0.303
2 - 4	INFLUENZA A	0	0	0	36.9	0	380.0	0.301
2 - 5	INFLUENZA A	0	0	0	36.3	37.1	400.3	0.317
2 - 6	INFLUENZA A	INFLUENZA A H1-2009	0	0	0	0	398.0	0.315
2 - 7	INFLUENZA A	INFLUENZA A H1-2009	0	0	0	0	385.7	0.306
2 - 8	INFLUENZA A	INFLUENZA A H1-2009	0	0	0	0	384.7	0.305
2 - 9	INFLUENZA A	INFLUENZA A H1-2009	0	0	0	0	407.0	0.322
2 - 10	INFLUENZA A	INFLUENZA A H1-2009	0	0	0	0	401.0	0.318
2 - 11	INFLUENZA B	0	0	29.4	0	0	408.7	0.324
2 - 12	INFLUENZA A	INFLUENZA A H1-2009	0	0	0	0	377.3	0.299
2 - 13*	INFLUENZA A	INFLUENZA A H1-2009	0	0	0	0	417.0	0.330

2 - 14	INFLUENZA A	INFLUENZA A H1-2009	0	0	0	0	416.0	0.330
2 - 15	INFLUENZA A	0	0	0	35.9	37.2	417.3	0.331
2 - 16	INFLUENZA A	0	0	0	36	0	404.7	0.321
2 - 17	INFLUENZA B	0	0	24.8	0	0	340.7	0.270
2 - 18	INFLUENZA A	0	0	0	36.6	38	406.3	0.322
2 - 19	INFLUENZA B	0	0	36.7	0	0	422.3	0.335
2 - 20	INFLUENZA A	0	0	0	31.4	33.9	413.7	0.328
2 - 21	INFLUENZA B	0	0	38.6	0	0	424.3	0.336
2 - 22	INFLUENZA B	0	0	35.3	0	0	401.7	0.318
2 - 23	INFLUENZA B	0	0	26.3	0	0	409.0	0.324
2 - 24	INFLUENZA B	0	0	24.3	0	0	410.3	0.325
2 - 25	INFLUENZA B	0	0	31.5	0	0	446.7	0.354
2 - 26*	INFLUENZA B	0	0	26	0	0	394.3	0.312
2 - 27*	INFLUENZA A	INFLUENZA A H3	INFLUENZA B	0	0	0	416.3	0.330
2 - 28	INFLUENZA B	0	0	23.4	0	0	431.7	0.342
2 - 29	INFLUENZA B	0	0	25.8	0	0	404.0	0.320
2 - 30*	INFLUENZA A	INFLUENZA B	0	18.2	30.3	30.4	382.3	0.303
2 - 31	INFLUENZA A	0	0	0	27.6	28.4	421.0	0.334
2 - 32*	INFLUENZA A	INFLUENZA A H1-2009	0	0	0	0	420.7	0.333
2 - NTC	N/A	N/A	N/A	N/A	N/A	N/A	440.7	0.349

* Samples chosen for SHINE-H5-LFA specificity validation

Supplementary Table 4. Primer, crRNA, and reporter sequences ordered through IDT.

Name	Oligo type	Sequence	Assays used
H5_Design1_RPA_T7_F	custom DNA oligo	GAAATTAATACGACTCACTATAGGGGAGAGTAATGGAAATTTCATTGCTCCAGAA	Prototype SHINE H5, SHINE-H5, SHINE-H5-LFA
H5_Dsign1_RPA_Re	custom DNA oligo	GGAATGGCATACTAGAGTTTATCGCCCCTA	Prototype SHINE H5, SHINE-H5, SHINE-H5-LFA
H5_Design1_RPA_nonT7_F	custom DNA oligo	GAGAGTAATGGAAATTTCATTGCTCCAGAA	SHINE-H5, SHINE-H5-LFA
H5_CladeSpecific_RPA_F	custom DNA oligo	GAAATTAATACGACTCACTATAGGGTTTCGAGAGTAATGGAAATTTCATTGCTCCA	2.3.4.4b clade-specific H5 SHINE
H5_CladeSpecific_RPA_R	custom DNA oligo	AATGGCATACTAGAATTTATCGCTCCTA	2.3.4.4b clade-specific H5 SHINE
H7_Eurasian_RPA_T7_F	custom DNA oligo	GAAATTAATACGACTCACTATAGGGTCAAACTAAGCAGCGGCTACAAAGATGTGA	SHINE-H7-Eurasian
H7_Eurasian_RPA_T7_R	custom DNA oligo	AGGCCCATTACAATGGCTAGAAGTATGAAA	SHINE-H7-Eurasian
H7_Eurasian_RPA_nonT7_F	custom DNA oligo	TCAAACTAAGCAGCGGCTACAAAGATGTGA	SHINE-H7-Eurasian
FAM-6U-Q reporter	custom RNA oligo	/56-FAM/rUrUrUrUrUrU/3IABkFQ/	Prototype SHINE H5, SHINE-H5, SHINE-H7-Eurasian
FAM-14U-Bio reporter	custom RNA oligo	/5BiosG/rUrUrUrUrUrUrUrUrUrUrUrUrUrU/36-FAM/	SHINE-H5-LFA, SHINE-H7-Eurasian-LFA
H5_Design1_crRNA	CRISPR Custom Guide RNA	/AltR1/rGrArUrUrUrArGrArCrUrArCrCrCrCrArArArArArCrGrArArGrGrGrGrArCrUrArArArArCrCrCrCrCrUrUrUrCrUrUrGrArCrArArUrUrUrUrGrUrArUrGrCrArUrA/AltR2/	Prototype SHINE H5, SHINE-H5, SHINE-H5-LFA
H5_Design2_crRNA	CRISPR Custom Guide RNA	/AltR1/rGrArUrUrUrArGrArCrUrArCrCrCrCrArArArArArCrGrArArGrGrGrGrArCrUrArArArArCrGrCrArGrUrArUrUrCrArGrArArGrArArGrCrArArGrArUrUrArArArA/AltR2/	Prototype SHINE H5
H5_CladeSpecific_Design1_crRNA	CRISPR Custom Guide RNA	/AltR1/rGrArUrUrUrArGrArCrUrArCrCrCrCrArArArArArCrGrArArGrGrGrGrArCrUrArArArArCrUrUrUrGrGrUrGrUrUrGrCrArGrUrGrGrCrCrArUrArUrUrCrCrArCrU/AltR2/	Prototype 2.3.4.4b clade-specific H5 SHINE
H5_CladeSpecific_Design2_crRNA	CRISPR Custom Guide RNA	/AltR1/rGrArUrUrUrArGrArCrUrArCrCrCrCrArArArArArCrGrArArGrGrGrGrArCrUrArArArArCrUrUrGrArCrArUrUrUrGrGrUrGrUrUrGrCrArGrUrGrGrCrCr	Prototype 2.3.4.4b clade-specific H5 SHINE

		ArUrArU/AltR2/	
H5_CladeSpecific_Design3_crRNA	CRISPR Custom Guide RNA	/AltR1/rGrArUrUrUrArGrArCrUrArCrCrCrCrArArArArArCrGrArArGrGrGrArCrUrArArArArCrUrUrGrGrUrGrUrUrGrCrArGrUrGrGrCrCrArUrArUrUrCrCrArCrUrC/AltR2/	Prototype 2.3.4.4b clade-specific H5 SHINE, SHINE-H5-CS
H7_Eurasian_crRNA	CRISPR Custom Guide RNA	/AltR1/rGrArUrUrUrArGrArCrUrArCrCrCrCrArArArArArCrGrArArGrGrGrArCrUrArArArArCrCrArUrGrArUrGrCrCrCrCrGrArArGrCrUrArArArCrCrArArArGrUrA/AltR2/	SHINE-H7-Eurasian

Supplementary Table 5. gBlock sequences ordered through IDT.

<table>
<tr><th>Name</th><th>Oligo type</th><th>Sequence</th><th>Assays used</th><th>Strain origin</th></tr>
<tr><td>H5_strain1_full</td><td>gBlocks Gene Fragment</td><td>GAAATTAATACGACTCACTATAGGGATGGAGAACATAGTACTTCTTCTTGC
AATAGTTAGCCTTGTTAAAAGTGATCAGATTTGCATTGGTTACCATGCAAA
CAATTCGACAGAGCAAGTTGACACGATAATGGAAAAGAACGTCACTGTTAC
ACATGCCCAAGACATACTGGAAAAAACACACAACGGGAAGCTCTGTGATCT
AAATGGGGTGAAGCCTCTGATTTTAAAGGATTGTAGTGTAGCTGGATGGCT
CCTCGGAAACCCAATGTGCGACGAATTCATCAGAGTGCCGGAATGGTCCTA
CATAGTGGAGCGGACTAATCCAGCTAATGACCTCTGTTACCCAGGGAGCCT
CAATGACTATGAAGAACTGAAACACCTGTTGAGCAGAATAAATCATTTTGA
GAAGATTCTGATCATCCCCAAGAGTTCCTGGCCAAATCATGAAACATCACT
AGGGGTGAGCGCAGCTTGTCCATACCAGGGAGCGCCCTCCTTTTTCAGAAA
TGTGGTGTGGCTTATCAAAAAGAACGATGCATACCCAACAATAAAGATAAG
CTACAATAATACCAATCGGGAAGATCTCTTGATACTGTGGGGGATTCATCA
TTCCAACAATGCAGAAGAGCAGACAAATCTCTACAAAAACCCAACCACCTA
CATTTCAGTTGGAACATCAACTTTAAACCAGAGGTTGGTACCAAAAATAGC
TACTAGATCCCAAGTAAACGGGCAACGTGGAAGAATGGACTTCTTCTGGAC
AATTTTAAAACCAGATGATGCAATCCATTTCGAGAGTAATGGAAATTTCAT
TGCTCCAGAATATGCATACAAAATTGTCAAGAAAGGGGACTCAACAATTAT
GAAAAGTGGAGTGGAATATGGCCACTGCAACACCAAATGTCAAACCCCAGT
AGGAGCGATAAATTCTAGTATGCCATTCCACAACATACATCCTCTCACCAT
TGGGGAATGCCCCAAATATGTGAAGTCAAACAAGTTGGTCCTTGCGACTGG
GCTCAGAAATAGTCCTCTAAGAGAAAAGAGAAGAAAAAGAGGCCTGTTTGG
GGCGATAGCAGGGTTTATAGAGGGAGGATGGCAGGGAATGGTTGATGGTTG
GTATGGGTACCATCATAGCAATGAGCAGGGGAGTGGGTACGCTGCAGACAA
AGAATCCACCCAAAAGGCAATAGATGGAGTTACCAATAAGGTCAACTCAAT
CATTGACAAAATGAACACTCAATTTGAGGCAGTTGGAAGGGAGTTTAATAA
CTTAGAAAGGAGGATAGAGAATTTGAACAAGAAAATGGAAGACGGATTCCT
AGATGTCTGGACCTATAATGCTGAACTTCTAGTTCTCATGGAAAACGAGAG
GACTCTAGATTTCCATGATTCAAATGTCAAGAACCTTTACGACAAAGTCAG
ACTACAGCTTAGGGATAATGCAAAGGAGCTGGGTAACGGCTGTTTCGAATT
CTATCACAAATGCGATAATGAATGTATGGAAAGTGTGAGAAATGGGACGTA
TGACTACCCTCAGTATTCAGAAGAAGCAAGATTAAAAAGAGAAGAAATAAG
CGGAGTGAAATTAGAATCAATAGGAACTTACCAGATACTGTCAATTTATTC
AACAGCGGCGAGTTCCCTAGCACTGGCAATCATGATGGCTGGTCTATCTTT
ATGGATGTGCTCCAATGGGTCGTTACAGTGCAGAATTTGCATCTAG</td><td>Prototype SHINE H5</td><td>|A/white-tailed_eagle/Estonia/2021|2021-05-16|1945381</td></tr>
<tr><td>H5_strain2_full</td><td>gBlocks Gene Fragment</td><td>GAAATTAATACGACTCACTATAGGGATGGAGAACATAGTACTTCTTCTTGC
AATAGTTAGCCTTGTTAAAAGTGATCAGATTTGCATTGGTTACCATGCAAA
CAATTCGACAGAACAGGTTGACACGATAATGGAAAAGAACGTCACTGTTAC
ACATGCCCAAGACATACTGGAAAAAACACACAACGGGAAGCTCTGTGATTT
AAATGGGGTGAAGCCTCTGATTTTAAAGGATTGTAGTGTAGCTGGATGGCT
CCTCGGAAACCCAATGTGCGACGAATTCATCAGAGTGCCGGAATGGTCCTA
CATAGTGGAGCGGGCTAATCCAGCTAATGACCTCTGTTACCCAGGGAGCCT
CAATGACTATGAAGAACTGAAACACCTGTTGAGCAGAATAAATCATTTTGA
GAAGATTCTTATCATCCCCAAGAGTTCTTGGCCAAATCATGAAACATCACT
AGGGGTGAGCGCAGCTTGTCCATACCAGGGAGCGCCCTCCTTTTTCAGAAA</td><td>Prototype SHINE H5</td><td>|A/mute_swan/England/328136/2022|2022-10-03|2198908</td></tr>
</table>

		TGTGGTGTGGCTTATCAAAAAGAACGATGCATACCCAACAATAAAGATAAG<br>CTACAATAATACCAATAGGGAAGATCTCTTGATACTGTGGGGGATTCATCA<br>TTCCAACAATGCAGAAGAACAGACAAATCTCTATAAAAACCCAACCACCTA<br>CATTTCAGTTGGAACATCAACTTTAAACCAGAGGTTGGTACCAAAAATAGC<br>TACTAGATCCCAAGTAAACGGGCAACGTGGAAGAATGGACTTCTTCTGGAC<br>AATTTTAAAACCAGATGATGCAATCCATTTCGAGAGTAATGGAAATTTCAT<br>TGCTCCAGAATATGCATATAAAATTGTCAAGAAAGGGGACTCAACAATTAT<br>GAAAAGTGGAGTGGAATATGGCCACTGCAACACCAAATGTCAAACCCCAGT<br>AGGAGCGATAAATTCTAGTATGCCATTCCACAACATACATCCTCTCACCAT<br>TGGGGAATGCCCCAAATACGTGAAGTCAAACAAGTTGGTCCTTGCGACTGG<br>GCTCAGAAATAGTCCTCTAAGAGAAAAGAGAAGAAAAAGAGGCCTGTTTGG<br>GGCGATAGCAGGGTTTATAGAGGGAGGATGGCAGGGAATGGTTGATGGTTG<br>GTATGGGTACCATCATAGCAATGAGCAGGGGAGTGGGTATGCTGCAGACAA<br>AGAATCTACCCAAAAGGCAATAGATGGAGTTACCAATAAGGTCAACTCAAT<br>CATTGACAAAATGAACACTCAATTTGAGGCAGTTGGAAGGGAGTTTAATAA<br>CTTAGAAAGGAGGATAGAGAATTTGAACAAGAAAATGGAAGACGGATTCCT<br>AGATGTCTGGACCTATAATGCTGAACTTCTAGTTCTCATGGAAAACGAGAG<br>GACTCTAGATTTCCATGATTCAAATGTCAAGAACCTTTACGACAAAGTCAG<br>ACTACAGCTTAGGGATAATGCAAAGGAGCTGGGTAATGGCTGTTTCGAATT<br>CTATCACAAATGCGATAATGAATGTATGGAAAGTGTGAGAAATGGGACGTA<br>TGACTACCCTCAGTATTCAGAAGAAGCAAGGTTAAAAAGAGAAGAAATAAG<br>CGGAGTGAAATTAGAATCAATAGGAACTTACCAGATACTGTCAATTTATTC<br>AACAGCGGCGAGTTCCCTAGCACTGGCAATCATGATAGCTGGTCTATCTTT<br>ATGGATGTGCTCCAATGGGTCGTTACAGTGCAGAATTTGCATTTAGATTTG<br>TGAGCTCAGATTGTAGTTAAAAACACCCTTGTTTCTACT		
H5_strain2_part	gBlocks Gene Fragment	GAAATTAATACGACTCACTATAGGGATCCCAAGTAAACGGGCAA<br>CGTGGAAGAATGGACTTCTTCTGGACAATTTTAAAACCAGATGA<br>TGCAATCCATTTCGAGAGTAATGGAAATTTCATTGCTCCAGAAT<br>ATGCATATAAAATTGTCAAGAAAGGGGACTCAACAATTATGAAA<br>AGTGGAGTGGAATATGGCCACTGCAACACCAAATGTCAAACCCC<br>AGTAGGAGCGATAAATTCTAGTATGCCATTCCACAACATACATC<br>CTCTCACCATTGGGGAATGCCCCAAATACGTGAAGTCAAACAAG<br>TTGGTCCTTGCGACTGGGCTCAGAAATAGTCCTCTAAGAGAAAA<br>GAGAAGAAAAAGAGGCCTGTTTGGGGCGATAGCAGGGTTTATAG<br>AGGGAGGATGGCAGGGAATGGTTGATGGTTGGTATGGGTACCAT<br>CATAGCAATGAGCAGGGGAGTGGGTATGCTGCAGACAAAGAATC<br>TACCCAAAA	SHINE-H5, SHINE-H5-LFA	|A/mute_swan/England/328136/2022|2022-10-03|2198908
H5_2.3.4.4b_strain1	gBlocks Gene Fragment	GAAATTAATACGACTCACTATAGGGAACCAGAGGTTGGCACCAAAAATAGC<br>TACTAGATCCCAAGTAAACGGGCAACGTGGAAGAATGGACTTCTTCTGGAC<br>AATCTTAAAACCAGATGATGCAATCCATTTCGAGAGTAACGGAAATTTCAT<br>TGCTCCAGAGTATGCATACAAAATTGTTAAGAAAGGGGACTCGACAATTAT<br>GAAAAGTGGAGTGGAATATGGCCACTGCAACACCAAATGTCAAACCCCAGT<br>AGGTGCGATAAATTCTAGTATGCCATTTCACAACATACATCCTCTCACCAT<br>TGGGGAATGCCCCAAATACGTGAAATCAAACAAGTTGGTCCTTGCGACTGG<br>GCTCAGAAATAGTCCTCTAAGAGAAAAGAGAAGAAAAAGAGGTCTGTTTGG<br>GGCGAAGCAGGGTTTATAGAGGGAGGATGGCAGGGAATGGTTGATGGTTGG<br>TATGGGTACCATCATAGCAATGAGCAGGGGAGTGGG	Prototype 2.3.4.4b clade-specific H5 SHINE, SHINE-H5-CS	|A/Texas/37/2024|2024-03-28|EPI3171488

H5_2.3.4.4b_strain2	gBlocks Gene Fragment	GAAATTAATACGACTCACTATAGGGCACCAAAAATAGCTACTAGATCCCAAGTAAACGGGCAACGTGGAAGAATGGACTTCTTCTGGACAATCTTAAAACCAGATGATGCAATCCATTTCGAGAGTAACGGAAATTTCATTGCTCCAGAATATGCATACAAAATTGTTAAGAAAGGGGACTCGACAATTATGAAAAGTGGAGTGGAATATGGCCATTGCAACACCAAATGTCAAACCCCAGTAGGTGCGATAAATTCTAGTATGCCATTTCACAACATACATCCTCTCACCATTGGGGAATGCCCCAAATACGTGAAATCAAACAAGTTGGTCCTTGCGACTGGGCTCAGAAATAGTCCTCTAAGAGAAAAGAGAAGAAAAAGAGGTCTGTTTGGGGCGATAGCAGGGTTTATAGAGGGAGGATGGCAGGGAATGGTTGATGGTTGGTATGGGTACCATCATAGCAATGAGCAGGGGAGTGGGTACGCTGCGG	Prototype 2.3.4.4b clade-specific H5 SHINE, SHINE-H5-CS	|A/dairy_cattle/Texas/2024|2024-03-20|EPI3158678
H5_non 2.3.4.4b_strain	gBlocks Gene Fragment	GAAATTAATACGACTCACTATAGGGCAAAAATAGCTACTAGATCCAAGGTAAACGGGCAAAGTGGAAGGATGGATTTCTTCTGGACAATTTTAAAACCGAATGATGCAATCAACTTCGAAAGTAATGGAAATTTCATTGCTCCAGAATATGCATACAAAATTGTCAAGAAAGGGGACTCAGCAATTATGAAAAGTGAATTGGAATATGGTAACTGCAACACCAAGTGTCAAACTCCAGTGGGGGCGATAAACTCTAGTATGCCATTCCACAACATACATCCTCTCACCATCGGGGAATGCCCCAAATATGTGAAATCAAACAGATTAGTCCTTGCGACTGGGCTCAGAAATAGCCCTCAAAGAGAGAGAAGAAAAAAAAGAGGACTATTTGGAGCTATAGCAGGTTTTATAGAGGGAGGATGGCAGGGAATGGTAGATGGTTGGTATGGATACCACCATAGCAATGAGCAGGGGAGTGGGTACGCTGCAGACAAAG	Prototype 2.3.4.4b clade-specific H5 SHINE, SHINE-H5-CS	|A/duck/Yunnan/5948/2003|2003-01-01|136318
H7Eurasian_strain	gBlocks Gene Fragment	GAAATTAATACGACTCACTATAGGGGTCCTATAATGCTGAACTCTTGGTGGCAATGGAGAATCAACACACAATTGACCTGGCAGACTCAGAAATGAACAAACTATACGAGCGAGTGAAAAGGCAACTGAGAGAGAATGCTGAAGAAGATGGCACTGGCTGTTTTGAAATATTCCACAAGTGTGATGACGACTGCATGGCCAGTATCAGAAACAACACTTATGATCACAGCAAATACAGGGAGGAGGCAATGCAAAACCGAATACAGATTAACCCGGTCAAACTAAGCAGCAGTTACAAAGATGTGATACTTTGGTTTAGCTTCGGGGCATCATGTTTCATACTTCTTGCCATTGCAATGGGCCTTGTCTTCATATGTGTGAAGAATGGAAACATGCGGTGCACTATTTGTATATAAGTTTGGAAAAAACACC	SHINE-H7-Eurasian	|A/chicken/Italy/1670/2015(H7N2)
H7American_strain	gBlocks Gene Fragment	GAAATTAATACGACTCACTATAGGGGTCGTATAATGCTGAACTGCTGGTAGCTATGGAAAATCAGCACACAATAGATCTTGCAGACTCAGAAATGAGCAAACTTTACGAGCGTGTAAGGAAACAACTGAGGGAGAATGCTGAAGAGGATGGGACTGGATGCTTTGAGATATTCCATAAGTGTGATGATCAATGCATGGAGAGCATAAGGAACAACACCTATGACCATACCCAATACAGAGCAGAGTCATTGCAGAATAGAATACAGATAGACCCAGTGAAACTGAGTAGTGGATACAAAGACATAATCTTATGGTTTAGCTTCGGGGCATCATGTTTTCTTCTTCTAGCCATTGCAATGGGATTGGTCTTCATTTGCATAAAGAATGGAAACATGCAGTGCACTATTTGTATATAGTTTGAGAAAAA	SHINE-H7-Eurasian	|A/mallard/New York/AH0081558/2016(H7N7)

Supplementary Table 6. Summary of final SHINE assay primer, crRNA and experiment condition.

**Name**	**Primer**	**crRNA**	**FAM 6rU quenched reporter**	**Relevant figures**
SHINE-H5	H5_Design1_RPA_T7_F (40nM)<br>H5_Design1_RPA_nonT7_F (120nM)<br>H5_Design1_RPA_T7_R (160nM)	H5_Design1_crRNA	78.5 nM	Figure 2B, C, D; Supplementary figure 5-9
SHINE-H5-CS	H5_CladeSpecific_RPA_F (120nM)<br>H5_CladeSpecific_RPA_R (120nM)	H5_CladeSpecific_Design3_crRNA	62.5 nM	Figure 3C, D, E; Supplementary figure 12
SHINE-H7-Eurasian	H7_Eurasian_RPA_T7_F (30nM)<br>H7_Eurasian_RPA_nonT7_F (90nM)<br>H7_Eurasian_RPA_T7_R (120nM)	H7_Eurasian_crRNA	62.5 nM	Figure 4C; Supplementary figure 13, 14
